# Psychiatric Advance Directives Under the Convention on the Rights of Persons With Disabilities: Why Advance Instructions Should Be Able to Override Current Preferences

**DOI:** 10.3389/fpsyt.2019.00631

**Published:** 2019-09-11

**Authors:** Matthé Scholten, Astrid Gieselmann, Jakov Gather, Jochen Vollmann

**Affiliations:** ^1^Institute for Medical Ethics and History of Medicine, Ruhr University Bochum, Bochum, Germany; ^2^Department of Psychiatry, Psychotherapy, and Preventive Medicine, LWL University Hospital, Ruhr University Bochum, Bochum, Germany

**Keywords:** psychiatric advance directives, advance statements, United Nations Convention on the Rights of Persons with Disabilities, substitute decision making, supported decision making, informed consent, competence, mental capacity

## Abstract

Psychiatric advance directives (PADs) are documents by means of which mental health service users can make known their preferences regarding treatment in a future mental health crisis. Many states with explicit legal provisions for PADs have ratified the United Nations (UN) Convention on the Rights of Persons with Disabilities (CRPD). While important UN bodies consider PADs a useful tool to promote the autonomy of service users, we show that an authoritative interpretation of the CRPD by the Committee on the Rights of Persons with Disabilities has the adverse consequence of rendering PADs ineffective in situations where they could be of most use to service users. Based on two clinical vignettes, we demonstrate that reasonable clinical recommendations can be derived from a more realistic and flexible CRPD model. Concerns remain about the accountability of support persons who give effect to PADs. A model that combines supported decision making with competence assessment is able to address these concerns.

## Introduction

Psychiatric advance directives (PADs) are documents that enable mental health service users to make known their preferences regarding treatment in a future mental health crisis. By now, many countries have explicit legal provisions for PADs. Examples are Australia, Belgium, Canada, Germany, Ireland, India, Scotland, The Netherlands, the United Kingdom, and various states in the United States. With the notable exception of the United States, all these states have ratified the United Nations (UN) Convention on the Rights of Persons with Disabilities (CRPD), adding up to a total of 177 ratifications of the convention to date (1).

The Committee on the Rights of Persons with Disabilities (the Committee) and other important UN bodies consider PADs as important instruments contributing to the realization of the convention’s general aims, such as promoting the autonomy and ensuring equal treatment of persons with disabilities (2–4). First efforts have been made to conceptualize PADs under the CRPD (5, 6). In the meantime, critics have raised the concern that the radical CRPD model developed by the Committee and adopted by other UN bodies will have the adverse effect of rendering PADs ineffective in typical mental health crises, thus depriving mental health service users of the opportunity to remain in control of their life and treatment by planning in advance (7–9).

By ratifying international human rights documents, states incur the obligation to incorporate the legal provisions of these documents in domestic law. If the critics are right, the legal provisions for PADs in countries that ratified the CRPD are thus at risk of being rendered ineffective. Thus far, it has not been clarified exactly why the radical CRPD model proposed by the Committee would seriously limit service users’ opportunities to plan their treatment in advance. The aim of this article is to determine whether the radical CRPD model promotes or impedes the realization of service users’ objectives in completing PADs.

The method for this paper is empirically and clinically informed conceptual and ethical analysis. After reviewing the empirical evidence on service users’ attitudes toward PADs, we proceed by outlining two different models of the informed consent process. We first delineate what we have elsewhere called “the combined supported decision making model” (8). Supported decision making in the mental health care informed consent process refers to interventions aimed at enhancing service users’ ability to make informed treatment decisions. The combined model combines supported decision making with an assessment of functional decision making capacities and with substitute decision making in cases where a person’s functional decision making capacities remain below the threshold of competence despite the provision of support. Then we present what might be called “the radical CRPD model.” This model is endorsed by several authoritative UN human rights bodies. According to the model, supported decision making should fully replace competence assessment and substitute decision making. After presenting two clinical vignettes, we derive clinical recommendations from each model. Here we argue that the combined supported decision making model promotes service users’ goals in completing PADs, whereas the radical CRPD model renders PADs ineffective in cases where they could be of most use to service users. A more realistic and flexible CRPD model is more conducive to the realization of service users’ goals but still raises concerns about the accountability of support persons. We close by giving recommendations for the implementation of PADs which can be supported by both the combined supported decision making model and the flexible CRPD model.

## Service Users’ Attitudes Toward PADs

Proponents of the radical CRPD model and proponents of progressive capacity-based models agree that far-reaching legal reform is necessary to promote the autonomy and ensure the equal treatment of mental health service users. Their disagreement is rather over whether the agenda for legal reform set by the radical CRPD model is conducive to the realization of these aims. This paper focuses specifically on the question whether the radical CRPD model promotes or impedes the realization of service users’ objectives in completing PADs. To be able to answer this question, it is crucial that the voices of service users be heard: why do they decide to complete a PAD and what are their expectations of this instrument? We will thus start by reviewing the empirical evidence on mental health service users’ attitudes toward PADs.

### Service Users’ Interest in PADs

Empirical studies consistently report a high interest in PADs among service users. A survey among 1,011 community mental health service users in five U.S. cities found that between 66% and 77% of respondents would want to complete a PAD if provided assistance (10). This study confirmed the findings of earlier studies with smaller sample sizes (11, 12). Research suggests that the interest in PADs is equally high in minority groups. A U.S. study among Latino service users using a structured interview found that 84% of 85 service users expressed interest in completing a PAD (13).

Comparable results were found in other countries. In a survey study among service users and clinicians conducted in New Zealand, 93% of 110 service users agreed that they supported PADs, and 87% indicated that they would participate in a PAD initiative if it were available (14). A survey study among 544 service users with bipolar disorder conducted in England and Wales found that 74% of respondents rated planning their care in advance as very important (15). A similar picture emerged in low- to middle-income countries with a more family-oriented approach to medical decision making. In a descriptive study conducted in India, 67% of 182 participants said they welcomed PADs in the initial interview, and 96% composed a PAD during the study period (16). Likewise, an Indian semi-structured interview study with 45 persons with severe mental disorders found that 89% of respondents were willing to complete a PAD (17).

The interest in PADS seems equally high among service users who have experienced involuntary commitment. In an Irish study, 84% of 67 service users who had been under involuntarily commitment expressed an interest in completing a PAD 1 year after discharge, even if only 56% believed that there are situations in which involuntary treatment with medication may be justified (18). Furthermore, research suggests that endorsement rates remain high after PAD completion. In a U.K.-based randomized controlled trial on joint crisis plans, 90% of the 44 participants in the intervention arm who were interviewed shortly after PAD completion said they would recommend the joint crisis plan to others, and 82% of 50 participants still held this view when interviewed at a 15 months follow-up (19).

The high interest in PADs among service users stands in stark contrast with the actual completion rates. A striking illustration of this is that in the aforementioned survey study in which between 66% and 77% of respondents indicated that they would want to complete a PAD, only 4% to 13% percent had a PAD (10). The low completion rates should not be interpreted as a lack of readiness among service users to complete a PAD. Studies found several barriers to PAD completion, such as lack of knowledge of, information about, and support for PADs (20–22). Research suggests that these barriers can be overcome by providing service users with support in completing PADs. A randomized controlled trial on facilitated PADs involving 469 persons with severe mental disorders found that 61% of participants in the intervention group completed a PAD after participating in facilitation sessions, compared to only 3% of participants in the control group (23).

### Service Users’ Attitudes Toward PADs

There is data available on service users’ reasons for their interest in PADs. One source are surveys carried out among service users irrespective of whether they have a PAD. In a recent survey study conducted in New Zealand, between 90% and 94% of 110 responding service users either agreed or strongly agreed to statements that PADs increase service users’ sense of responsibility, empowerment, and autonomy (14). This study confirmed earlier findings. In a U.S. survey study among various stakeholders, 82% of 104 persons with schizophrenia agreed or strongly agreed to the statement that PADs will give them more control over their own lives and over what happens to them in the future (11). In another survey among stakeholders conducted in the U.S., 87% of 32 responding service users agreed to the statement that psychiatric advance directives will help people with mental disorder feel more in control of their lives; in addition, the theme of empowerment, control, and rights was most prevalent in answers to open-ended questions on the benefits of PADs (24). The authors quote the following exemplary answer from a service user:

“I think psychiatric advance directives will help persons with mental illness feel some measure of control in their lives because they will be participating in big decisions concerning their lives. They will make the illnesses more real. Patients are taking responsibility for their well-being/recovery.”

There are also data available on whether service users still see empowerment and control as benefits of PADs after they completed a PAD and subsequently experienced a mental health crisis. A quantitative intervention study on facilitated PADs for persons with psychotic disorders did not find a general improvement of perceived treatment self-determination as compared to baseline scores at a follow-up 12 months after PAD completion (25). In other studies, the rates of reported control and empowerment remained high in the course of the study. In the context of a randomized controlled trial on joint crisis plans, 71% (n = 32) of participants who completed a joint crisis plan reported that they felt more in control of their mental health problem at immediate follow-up, and 56% (n = 28) of the participants held this view at a follow-up at 15 months (19). An interview study conducted in the U.S. similarly found that 26 of 30 service users approved of PADs at baseline, and 23 of 27 service users stated empowerment as the reason for their approval (26). Here, too, the initial enthusiasm waned somewhat in the course of the study: 23 of 26 service users who completed a PAD still agreed or agreed strongly that they are satisfied with the PAD, but 12 of them also voiced a variety of concerns.

A possible explanation of the discrepancy between feelings of empowerment at baseline and at follow up is the failure of the clinical team to consider and respect PADs fully. Clinicians may first of all fail to access the PAD because they are unaware of its existence or because it is unavailable at the time of a mental health crisis. Moreover, in many jurisdictions, clinicians must merely take into account a PAD as a source of information for medical decision making and may override it when doing so is judged to be in the service users’ medical best interest. Improvements in both policy and practice are thus called for.

A U.S.-based interview study with service users with psychotic disorders who experienced a mental health crisis after PAD completion suggests that service users tend to consider PADs valuable even when they are not consistently followed by the clinical team. In this study, self-determination and empowerment emerged as one of three major themes (27). One service user explained:

*“Yes, I want control even if I’m not in control. You know? Control issues are always an issue for me when an emergency occurs and if I need to be admitted to a hospital and give up controls, then I still want people to know what’s best for me.”*

During the interviews, many participants complained that their PADs were not consulted or honored during mental health crises. Nevertheless, most of them still approved of the instrument, with one participant giving the following exemplary explanation:

*“It’s probably one of the best things that’s come into mental health in a long time because it gives you rights, while you’re sound and while you know what’s best for you – and you’re the only person that knows what’s best for you deep down. [ … ] at least this way you do have some say in your treatment if it’s read and people see it and it’s legal.”*

In cultures with a more family-oriented approach to medical decision making, service users tend to consider empowerment as an important benefit of PADs as well. In an interview study conducted in India, 16 of 18 service users who completed a PAD were happy at being offered the opportunity to document their own wishes and preferences regarding future treatment (28). The authors quote a female service user:

*“I think writing the PAD will help me have control over future treatment, because I wrote it like a will, for my safety in the future. I liked it and think it will help me have control.”*

Empirical evidence from quantitative studies suggests that service users’ perception that PADs enable them to stay in control of their care is not merely subjective. A study conducted in the U.S. showed that across 90 crisis events in which a PAD was accessed by the treatment team, the average rate of care consistent with the instructions in the PAD was 67%, which is comparable with the consistency rate for advance directives for somatic care (29).

### The Content of PADs

The content of PADs provides a good indication of service users’ goals in completing them. Given that PADs were developed originally in the anti-psychiatric movement as a protection from psychiatry (30), a common concern is that service users will use PADs to refuse all psychiatric treatment in advance (31). Although promotion campaigns and PAD templates of some service user organizations give some cause for this concern (32, 33), empirical research showed that the seriousness of the issue should not be overestimated. Of a total number of 402 PADs analyzed in studies carried out in the U.S., the U.K., and India, none contained a general refusal of psychiatric treatment (23, 26, 34–36).

Another common concern is that PADs may contain ambiguous instructions for medical decision making. Research has shown this worry to be unfounded as well. In one study, physicians rated the instructions as feasible and consistent with practice standards for at least 95% of the 106 PADs (36). Another study similarly found that between 83% and 94% of 136 analyzed PADs contained preferences that were rated as feasible and consistent with practice standards (23).

Studies that analyzed the content of PADs found that service users use them to document a variety of preferences regarding their treatment broadly conceived. Preferences regarding medical treatment form a central domain. In a study carried out by Srebnik and colleagues, 106 PADs of users of community mental health services were analyzed: 81% of PADs contained advance consent to treatment with specific medication, 64% an advance refusal of specific medications, and 72% an advance refusal of electroconvulsive therapy (ECT) (36). A study conducted by Swanson and colleagues in which 136 PADs were analyzed yielded similar results: 93% contained advance consent to treatment with at least one specified psychotropic medication, and 77% contained an advance refusal of some specified medications (23). In both studies, none of the analyzed PADs contained general refusal of psychiatric treatment.

The results of these two studies may be biased toward treatment because the service users included in these studies are likely to be well-integrated in the psychiatric system and completed the PADs in groups with the help of a peer trainer and special software, or in facilitated sessions involving semi-structured interviews and guided discussions. Reilly and Atkinson analyzed 55 PADs presented to the Mental Health Tribunal in Scotland (35). This sample may be biased toward treatment refusal, as cases that come before the court are likely to reflect conflicts between service users, substitute decision makers and mental health professionals. In this study, 45% of the analyzed PADs contained advance consent to specified medications, 53% contained advance refusals of specific medications, and 42% contained advance refusals of ECT. Again, no PAD contained an advance refusal of all psychiatric treatment. Although the rate of PADs containing advance consent to medical treatment found in this study was lower than in the other studies, the study confirms that service users use PADs to make known preferences among treatment options rather than to refuse psychiatric treatment altogether.

Hospital admission is another important domain. Although Srebnik and colleagues found that 68% of analyzed PADs documented a preference for alternatives to hospitalization over hospitalization, nearly all respondents recognized the need for hospitalization, with 80% of PADs specifying preferred hospitals in case of admission and 48% specifying hospitals to avoid (36). The study carried out by Swanson and colleagues yielded comparable results: 89% of analyzed PADs contained an advance agreement to hospitalization in at least one inpatient facility, while 61.8% contained advance refusals of admission to particular hospitals (23).

Service users also use PADs to appoint substitute decision makers: 46% of participants in the study conducted by Srebnik and colleagues and around 77% in the study conducted by Swanson and colleagues appointed a substitute decision maker in their PAD (23, 36). Research shows that the involvement of a substitute decision maker increases the likelihood that care is consistent with PAD instructions (29). Note that this finding should not be interpreted as counting in favor of substitute as compared to supported decision making, for it seems plausible to assume that the involvement of support persons or decision making assistants would have a comparable effect. Other things that service users document in their PADs are preferences regarding de-escalation methods and coercive measures as well as treatment-neutral preferences, such as instructions regarding contact persons, persons not authorized to visit during hospitalization, and care of finances, dependents, or pets (36).

A comparable picture emerged from recent studies conducted in India, suggesting that the content pattern of PADs delineated above is not unique to Western and high-income countries (16, 17, 34).

## The Combined Supported Decision Making Model

The normative status of PADs varies across models of the informed consent process. We will discuss two models, starting with the combined supported decision making model. This model builds on an influential model of informed consent process developed in medical ethics (37) and extends it to include supported decision making.

Respect for autonomy is a central normative pillar of the model, where autonomy is understood not so much as theability to do what one wants at a given point of time as the ability to shape one’s life according to one’s own conception of the good (8). The value of autonomy is recognized in three ways. First, the model recognizes the instrumental value of autonomy: in a society characterized by value pluralism, service users themselves are typically in the best position to assess which of the treatment options maximally promotes their well-being. Second, it recognizes the inherent value of autonomy: irrespective of one’s well-being, independently shaping one’s life in accordance with one’s own conception of the good is valuable in itself. The instrumental and inherent value of autonomy ground health professionals’ duty to respect treatment refusals of service users who are competent to consent and to abide by their positive treatment choices as long as these are compatible with practice standards. Third, the model recognizes service users’ positive claim on health professionals to be enabled to make autonomous choices. This grounds a duty on the part of health professionals not only to disclose the information about the consequences of the various treatment options in an understandable way but also to enhance service users’ decision making abilities by means of supported decision making.

When service users have difficulties grasping the expected consequences of their choices, as may happen in a mental health crisis, the combined model requires mental health professionals to provide decision support. Although supported decision making as such has a broader scope, in the context of the mental health care informed consent process, it refers to all types of interventions aimed at enabling service users to make informed treatment decisions. Examples are everyday interventions (e.g., giving time to adapt or providing tranquil surroundings), medical interventions (e.g., reducing sedative medication or treating dehydration and infection), interventions that improve the quality of the disclosure information (e.g., enhanced consent procedures), or interventions that facilitate communication (e.g., plain language, braille or sign language). Social support is another dimension. Notable examples are support from family or friends, peer support, and advocacy (8). Though tested primarily in the context of research consent, empirical studies showed that relatively simple interventions can enhance consent capacity substantially (38).

The aim of supported decision making in the mental health care informed consent process is to assist service user in decision making and to enable them to make informed treatment choices. The combined supported decision making model recommends using a functional competence assessment to assess whether this aim has been attained (8). The functional criteria for competence developed by Grisso and Appelbaum can be used to assess whether supported decision making suffices to enable service users to make informed treatment choices. On this functional approach, service users are competent (i.e., have mental capacity) to make a treatment decision if and only if they are able to understand the relevant information, appreciate that this information applies to their own situation, rationally process the information, and express a treatment choice (39).

A competence assessment takes about 20 to 30 min. It consists of a semi-structured conversation between service user and mental health professional in which the mental health professional discloses the information relevant to the treatment decision at hand in an understandable way and asks focused questions to probe whether the service user is able to understand and appreciate the information, evaluate the consequences of the treatment options in light of her values and commitments, and communicate a treatment choice (39, 40). Support tools can and should be used during this conversation to enhance service users’ decision making capacity.

On the functional approach, competence is a threshold concept defined in terms of a relevant threshold of functional decision making abilities. The concept is binary because it is designed to enable us to answer the practical question of whether persons should be allowed to decide for themselves or whether recourse should be taken to substitute decision making (41). Research shows that competence can be assessed in a non-arbitrary way, with very high levels of agreement among evaluators (42). The functional approach is furthermore non-discriminatory, as the criteria apply to all persons, regardless of whether they have a mental disorder. Though mental disorder is a risk factor for incompetence (40), research showed that many persons with mental disorders are competent to make informed treatment decisions (43), while a substantial share of persons without mental disorders are not able to do so (44). Finally, on the functional approach, determinations of incompetence are valid only at a specific point of time and for a specific treatment decision, and hence there is no place for indefinite or plenary guardianship on this approach.

During a mental health crisis, the provision of support is sometimes insufficient to enable service users to evaluate the risks and benefits of the treatment options in light of their values and commitments and give valid consent. When unable to consent, service users sometimes make choices that are incompatible with their own conception of the good and which disrupt their life plans. Since the combined supported decision making model serves to protect and promote service users’ ability to shape their life according to their own conception of the good, it yields that service users’ current preferences should not carry decisive authority under the assumed conditions.

The German legal framework employs the term “free will” to refer to the preferences of a person who is able to give valid consent and the term “natural will” to refer to the preferences of a person who is unable to give valid consent (45). Recently, George Szmukler has interpreted the central CRPD notions of “will” and “preferences” in an analogous way (9, 46). The point can thus also be put as follows: the combined supported decision making model serves to protect and promote service users’ “will” or “free will” and thus grants the latter priority over service users’ “preferences” or “natural will.”

It is important to make explicit at this point that the combined model is fully capacity-based and does not permit substituted decision making based on the presence of mental disorder in combination with a perceived risk to self or others. When service users are unable to consent, the model introduces substitute decision making as a proxy for concurrent autonomous decision making. A substitute decision maker should thus decide *on behalf of* the service user who is unable to give consent. To give effect to the service user’s “will,” the substitute decision maker must make the treatment decision that the service user would make if she were competent to consent. The reason is that under the assumed conditions, these counterfactual preferences will better match service users’ own conception of the good than their current preferences. This does not mean, however, that current preferences can be ignored or set aside. Service users who are unable to give valid consent must be involved in the substitute decision making to the extent of their ability, and their preferences should be given careful consideration; it is only that these preferences are not as decisive as the current preferences of a person who is competent to consent.

The proposed standard for substitute decision making is commonly referred to as the “substituted judgment standard” (39–41). It can be contrasted with the so-called “best interest standard,” which requires substitute decision makers to make the treatment decision that is in the best interest of the service user. Although the best interest standard can be interpreted in a more subjective and person-oriented way, taking into account service users’ will and preferences (9), in clinical practice, it is often interpreted according the medical model as prescribing the treatment that is medically indicated. The two standards come apart because what a service user would want had she been competent to consent need not be in her best interest (or what others take to be her best interest). While in most jurisdictions the law employs the best interest standard, in some jurisdictions substitute decision makers must abide by the substituted judgment standard. Notable examples are Germany (45) and various states in the U.S. (40).

Substitute decisions must be based on concrete evidence rather than speculation. Medical ethicists have proposed that substitute decision makers must base their decision on the following types of evidence (8, 37, 39):

1. advance directive
2. previously expressed treatment preferences
3. service user’s values and commitments
4. service user’s best interest

The logic of the order is epistemic: a PAD provides the most reliable evidence of what the service user would want if she were competent to consent, followed by orally expressed treatment preferences at a time when the service user was competent to consent, and treatment preferences derived from the service user’s values and commitments. The notion of best interest in the last item on the list differs from that in the Mental Capacity Act (2005) in England and Wales. Where in the Mental Capacity Act, the notion of best interest functions as an independent normative standard for substitute decision making and encompasses other items on the above list, here it is construed narrowly and functions as a source of evidence: if there is absolutely no information available about a person’s beliefs, values, and preferences and treatment cannot be postponed, as may happen in an emergency situation, providing treatment as medically indicated is most likely to be in accordance with what the person would have wanted had she been competent. This situation is unlikely to occur in psychiatry.

The items are listed in order of priority. This means that substitute decision makers may proceed to the next item on the list only if the previous item is unavailable, unclear, or open to multiple interpretations. An important implication of this is that an unambiguous and specific treatment refusal contained in a PAD must be respected even if doing so is allegedly not in the service user’s best interest. In most jurisdictions, by contrast, the law leaves mental health professionals a lot of leeway to override PADs in what they take to be the best interest of service users. The combined supported decision making model challenges this approach. German guardianship law is a notable exception in this respect. If a person has a PAD, then according to the German Civil Code Section 1906a, para. 1 No. 3 BGB, involuntary treatment is permissible only if the treatment is compatible with the PAD (33).

On the combined supported decision making model, PADs fall under substitute decision making. Although there are other ways to spell out their legal force, on the combined model PADs depend on the concept of competence or mental capacity in two ways: PADs are valid only if service users are competent to write a PAD at the time of completion, and PADs enter into application if, and only if, service users are unable to make the treatment decision at hand. On the model, then, competence is a necessary condition for the validity of PADs and incompetence marks the point at which PADs take effect.

PADs can be either “binding” or “guiding.” Binding PADs are authoritative directives that directly bind substitute decision makers and mental health professionals. Consequently, binding PADs must be *respected* by substitute decision makers and health care professionals. Guiding PADs are authoritative sources of information about the preferences that service users would have in the circumstances if they were competent to consent. Accordingly, guiding PADs must be *considered* by substitute decision makers and health care professionals. Given that PADs are the first item on the prioritized list of grounds for substitute decisions, substitute decision makers and mental health professionals must normally give effect to the preferences in guiding PADs. Whether a given PAD is guiding or binding depends among other things on the legal context and the quality of its instructions. On the combined model, both types of PAD fall under substitute decision making inasmuch as they provide a basis for decision making that is different from, and hence potentially in conflict with, the current preferences of service users.

## The Radical CRPD Model

Important UN bodies have a positive attitude toward PADs and advance care planning more broadly conceived. The Committee, for example, claims that “all persons with disabilities have the right to engage in advance planning and should be given the opportunity to do so on an equal basis with others” (2). Other UN bodies share this view. The High Commissioner claims that “instruments such as advance directives or powers of attorney should be promoted” (4) and the UN Special Rapporteur on the rights of persons with disabilities includes advance directives under a number of “supported decision making regimes” that “states must develop [ … ] and intensify” (3).

Recall that on the combined supported decision making model, PADs function within a substitute decision making framework. The aforementioned UN bodies, on the other hand, call for the abolishment of substitute decision making regimes and their replacement by supported decision making arrangements (2–4). The Committee, for instance, claims that states parties have an “obligation to replace substitute decision making regimes by supported decision making,” warning us that supported decision making “should never amount to substitute decision making” (2). It is important to note that dissenting opinions on this issue can be found within the UN human rights framework. Notably, the Human Rights Committee and the Subcommittee on Prevention of Torture and Other Cruel, Inhuman or Degrading Treatment or Punishment do not support an absolute ban on substitute decision making and involuntary treatment (9, 47). Our focus in this section is on the radical abolitionist model. For ease of exposition, we will refer to this model as the “radical” CRPD model.

The call for the abolishment of all substitute decision making regimes is premised on a universal recognition of legal capacity. Legal capacity divides into legal standing and legal agency. Where legal standing denotes the ability to hold legal rights and duties, legal agency refers to the capacity to exercise those rights and duties, which can be done by entering into contracts and making legal transactions (2). According to the Committee, “all people, including persons with disabilities, have legal standing and legal agency simply by virtue of being human” (2). Since the act of giving informed consent involves an exercise of legal agency, the universal recognition of legal capacity entails a universal recognition of the right to give informed consent and to have one’s treatment decisions respected.

In keeping with this, the Committee rejects the functional approach to competence and holds that “all persons, regardless of disability or decision making skills, inherently possess legal capacity” (2). From this, it follows that it is impermissible to take recourse to substitute decision making when a competence assessment attests that a person’s functional decision making capacities fall below the relevant threshold of competence. The Committee rejects competence assessment and substitute decision making because it takes these practices to be discriminatory against persons with mental disabilities. Admitting that service users with substantially impaired decision making abilities sometimes make choices that are incompatible with their own conception of the good, the Committee nevertheless concludes that “at all times, including in crisis situations, the individual autonomy and capacity of persons with disabilities to make decisions must be respected” (2).

From this, it follows that service users’ current preferences prevail and, hence, that treatment preferences delineated in PADs may not be prioritized over service users’ current preferences. As a result, PADs are neutralized whenever they conflict with current preferences. Here, it is immaterial whether the PADs are binding or guiding. In the case of binding PADs, any current expression of preferences in conflict with those contained in the PAD involves an exercise of legal capacity and thus amounts to a formal revocation of the PAD. In the case of guiding PADs, both the preferences contained in the PAD and the person’s current preferences provide evidence for the person’s will and preferences, but there is no reason to assign more weight to past preferences documented on paper than to preferences currently voiced by service users.

Let us assess whether the relevant UN bodies accept these implications. The Special Rapporteur on the rights of persons with disabilities refers to advance directives as a supported rather than a substituted decision making arrangement. “Contrary to substitute decision making regimes,” she continues to explain, “under a supported decision making arrangement, legal capacity is never removed or restricted; [ … ] support must be provided based on the will and preferences of the individual” (3). The High Commissioner makes the point more explicit: “Even when such instruments [advance directives or powers of attorney] are in force,” he claims, “persons with psychosocial disabilities must always retain their right to modify their will and service providers should continue to seek their informed consent” (4). The Special Rapporteur and the High Commissioner thus endorse the view that service users’ current preferences constitute a revocation, or at least a modification, of their PAD whenever they conflict with the PAD instructions.

Accordingly, on the radical CRPD model, PADs seem to have legal effect only when service users are not able to express any wishes and preferences at all or when their current preferences are so diffuse that a sufficiently coherent interpretation of them is not possible. This indeed seems to be the Committee’s official position. “The ability to plan in advance is an important form of support,” it writes, “whereby [persons with disabilities] can state their will and preferences which should be followed at a time when they may not be in a position to communicate their wishes to others” (2). Likewise, the Special Rapporteur on the rights of persons with disabilities claims that advance directives “can be followed at a time when they may not be in a position to communicate [their will and preferences]” (3).

In view of this, the radical CRPD model of advance directives serves persons with mental disability only in a limited way. When in a coma, persons are not able to communicate their will and preferences, and the same may hold for persons in the late stage of dementia. Persons with moderate dementia may be able to express preferences, but these may be so diffuse that they cannot be interpreted in an unambiguous way. The radical CRPD model enables persons to plan in advance for such situations and so to remain in control of their treatment. But it is different with mental health crises. In a mental health crisis, service users typically remain able to communicate their preferences, and these preferences tend to be pronounced and unambiguous. It is quite common, for instance, that despite the support offered, service users in a mental health crisis tell others that they would rather be out on the street than be admitted to hospital. On the radical CRPD model, such pronounced preferences must be interpreted as a revocation of the PAD.

The empirical evidence on service users’ attitudes to PADs reviewed in this article suggests that service users show interest in PADs not because they anticipate situations in which they are unable to express their preferences with sufficient clarity, but because they anticipate situations in which they express clear and strong preferences that are incompatible with their deeply held values and commitments. By limiting the scope of application of PADs to conditions in which persons are unable to express their preferences with sufficient clarity, the CRPD model renders PADs ineffective in situations in which they can enable service users to remain in control of their life and treatment.

In closing this section, we must consider a proposal made by the Committee to account for the legal effect of PADs without dependence on the notion of competence or mental capacity. The Committee proposes that “the point at which an advance directive enters into force (and ceases to have effect) should be decided by the person and included in the text of the directive; it should not be based on an assessment that the person lacks mental capacity” (2). In this way, service users can authorize mental health professionals to enforce their PADs against their current preferences. The proposal is thus roughly to conceptualize PADs in general as so-called competence-insensitive self-binding directives, or Ulysses contracts. Other proponents of the radical CRPD model have endorsed the proposal (3, 48), but thus far, it has not been worked out beyond this rudimentary idea. Competence-insensitive self-binding directives raise a range of serious conceptual and ethical issues (49) which cannot be discussed within the limits of this paper. We shall therefore only briefly indicate why we are not convinced that the proposal is able to solve the problem delineated above. First, research suggests that the type of self-binding directive that can count on most support from service users is competence-sensitive (50, 51). Second, we think that on the radical CRPD model, it will be hard to explain why pronounced current preferences incompatible with instructions in a self-binding directive should not count as a revocation of the directive. These issues must be addressed more fully at another occasion.

## Conflicts Between Advance Instructions and Current Preferences: Two Case Vignettes

The theoretical disagreement between the two models and their implications for the normative status and effectiveness of PADs is clear, but it remains to be determined to what extent the two models yield different recommendations for clinical practice.

To be able to derive clinical recommendations, we will present two hypothetical case vignettes involving PADs. The vignettes are based on our clinical experience. They involve a service user with schizophrenia and a service user with bipolar disorder, as members of these diagnostic groups are likely to be affected by involuntary measures. The content of the PADs is based on the general pattern emerging from the empirical literature. To reflect the broad range of PAD instructions, we describe one PAD containing preferences regarding medical treatment and one PAD containing treatment-neutral preferences. The vignettes reflect a conflict between the person’s PAD instructions and the person’s current preferences. Such conflicts occur regularly in clinical practice and allow us to make explicit the differences between the two models.

### Case Vignette 1

Daniel, a 23-year-old man with a diagnosis of schizophrenia, is involuntarily admitted to a psychiatric hospital. The emergency services and police report that they were called by the man’s family after he had repeatedly bashed his head against the wall in a state of acute agitation. The incident occurred in the apartment where Daniel lives together with his parents and his sister, who is 2 years younger.

Daniel’s sister is present at the admission and tells the psychiatrist that she thinks her brother stopped taking his oral medication of 20-mg olanzapine a couple of weeks ago. He told his family that he believed the medication, though essential to treating psychotic episodes, cannot prevent future episodes. She tells the psychiatrist that in the last couple of weeks her brother became increasingly socially withdrawn, talked little to the rest of the family and spent most of the time alone in his room. Judging from the noises she heard from his room, she inferred that her brother slept little at night, and she also noted that he regularly refused to eat and drink, telling the family that he had no appetite.

During this conversation, Daniel sits quietly at his sister’s side. He is fairly cooperative and answers most of the questions during the admission interview, though he appears suspicious and avoids eye contact with the psychiatrist. In the psychiatrist’s judgment, Daniel has an acute psychotic episode with formal thought disorder, persecutory delusions, and auditory hallucinations, probably triggered by the abrupt stop of medication. She recommends Daniel to restart taking antipsychotic medication, explaining that this is likely to reduce his feelings of anxiety and state of agitation. Daniel suddenly jumps up from his chair and interrupts the psychiatrist, shouting: “I will never take anything from you, I know that you want to poison me!” Attempts to calm Daniel down and enter into a conversation with him fail, and he continues to refuse medication as well as other treatment offers made by the psychiatrist.

Thereupon, Daniel’s sister reaches for her purse and hands over an advance directive to the psychiatrist. She tells the treatment team that her brother composed the advance directive shortly after his last inpatient stay somewhat over a year ago. The advance directive contains the following instruction: “In case I become psychotic again, I do not want to be treated with typical antipsychotic drugs, such as haloperidol, because I have experienced many unpleasant side effects of these drugs in the past. I get the most out of a low dose therapy with atypical antipsychotics, such as olanzapine or quetiapine.”

### Case Vignette 2

Debby is a 46-year-old woman with bipolar disorder who is treated in a psychiatric hospital on a voluntary basis. The reason for her admission in the week before was that some of her best friends had noticed signs of a beginning manic episode: Debby was euphoric, talked a lot, slept poorly, and displayed an increased drive to engage in activities. Two friends shared their worries with her in a conversation at home and proposed to bring her to the psychiatric hospital. Debby agreed, though somewhat reluctantly. She doesn’t like staying in the hospital, but she benefited from inpatient treatments in earlier mental health crises and knows many of the mental health professionals working in the hospital.

During one of her previous inpatient stays, Debby had worked out a joint crisis plan with the support of a psychiatrist and a social worker. Among other things, the joint crisis plan contains statements about people with whom she wants to have contact during a manic episode and people with whom she wants to avoid contact. The latter group includes people from work but especially mentioned is her ex-husband Jason. Debby and Jason had been involved in what Debby described to her friends as an abusive relationship. Although they are divorced for some years now, they live in the same neighborhood and visit each other in irregular intervals. Sometimes these visits are enjoyable, but most of the time they end up in serious quarrels.

During the second week of the inpatient treatment, Debby’s mental health worsens and her symptoms increase. Euphoria switches to dysphoria and her thoughts become increasingly incoherent. Intermittently, she is very agitated and easily irritable and refuses to take medication, telling the treatment staff that she is going through some serious struggles and has other things to attend to. During the visit of the psychiatrist, Debby tells the psychiatrist that Jason called her on her cell phone and that he will visit her tonight. The treatment team reminds her of her joint crisis plan and recommends her to cancel the visit. Thereupon, Debby gets furious and exclaims: “Do you want to keep me from seeing Jason? I am not ill! If you don’t let him in, I’m out of here!”

## Clinical Implications of the Combined Supported Decision Making Model

The combined supported decision making model requires mental health professionals to provide support when it is reasonable to believe that service users’ functional decision making abilities are (temporarily) impaired. There are reasons to think that Daniel and Debby fail to understand and appreciate the nature and potential consequences of the available treatment options (including the option of no treatment). Believing that the psychiatrist wants to poison him, Daniel seems insufficiently able to see that treatment with atypical antipsychotics was helpful in treating previous psychotic episodes. Debby, on the other hand, seems insufficiently able to appreciate her own mental condition and to estimate the potential negative impact of seeing her ex-husband. The treatment team must provide support to enable Daniel and Debby to make an informed treatment decision. Which form of support is appropriate highly depends on the individual and the context. Concretely, we think that Daniel could profit from giving him time to put his mind at rest and involving a peer support worker in the admission process. For Debby, it could be helpful to contact a friend who can support her and knows about her difficult relationship with her ex-husband.

If the provision of support raises Daniel’s and Debby’s functional decision making capacities up to the point at which they are competent to make an informed treatment decision, Daniel and Debby can make their own decisions. We think that, if provided adequately, supported decision making can yield this result in many cases. To be able to make explicit the differences between the two models, however, we will assume for the sake of the argument that, despite the provision of support, Daniel and Debby remain insufficiently able to understand and appreciate the implications of the decision they face. The combined supported decision making model yields that a substitute decision must be taken in such cases.

On the combined model, substitute decision making must be guided by the substituted judgment standard and, hence, the preferences that service users would have if they were able to make an informed decision should guide decision making. In both vignettes, the PAD is the most authoritative source of evidence for these preferences. When competent to make an informed treatment decision, Daniel concluded that he wanted to be treated with olanzapine or quetiapine rather than haloperidol in case of a mental health crisis. Similarly, Debby reached the considered judgment that it would be better for her not to be in contact with her ex-husband when in hospital. Regardless of whether the PADs in question are binding or guiding, substitute decision makers and mental health professionals have strong reason to abide by the PADs on the combined model.

The recommendation that follows is that the clinical team should intervene, where the intended outcome of the intervention is that Daniel takes his preferred medication and that Jason does not visit Debby. The answer to the question how the clinical team should intervene is highly context-dependent. The general principle is that mental health professionals should take the least restrictive means to give effect to the PAD and ensure that the risks and burdens of the intervention are clearly outweighed by the expected benefits of its success.

We are inclined to think that using physical compulsion to administer medication to Daniel does not satisfy these prerequisites and that the same holds true for denying Jason access to the hospital. In Daniel’s case, the expected benefits of treatment with anti-psychotic medication do not immediately seem to outweigh the potential psychological harms of being subjected to physical compulsion and involuntary treatment. Moreover, it would seem that less restrictive alternatives are available. Various argumentative strategies, or “treatment pressures,” have been identified by means of which clinicians can guide service users toward a certain treatment option (52). It would thus be an option to temporarily break off the admission interview, give Daniel the opportunity to calm down, and try to convince him of the benefit of treatment in accordance with the PAD at a later point of time. The option of applying physical compulsion should be contemplated only as a last resort when all less restrictive strategies fail and the conditions in the above-stated general principle are fulfilled.

In Debby’s case, denying Jason admission to the hospital could result in an escalation of the situation and might have the unwanted effect of Debby leaving the hospital and visiting Jason of her own accord. It would thus seem that in this case, too, it is preferable to adopt transparent communicative strategies to convince Debby and Jason of the desirability of postponing their meeting. Should these strategies ultimately fail, the clinical team might attempt to arrange a visit of Jason under the supervision of a trusted member of the clinical team. Although this would compromise Debby’s PAD instructions, under the assumed circumstances the option seems more faithful to the instructions than any of the alternatives.

It goes without saying that none of these interventions would be appropriate if Daniel and Debby were competent to make the treatment decision at hand. On the combined supported decision making model, all aforementioned interventions fall under substitute decision decision making and, hence, must be guided by the decisions service users would make in the circumstances if they were competent to consent. It should be emphasized that the combined model does not favor treatment over non-treatment. After all, had Daniel’s PAD contained a general refusal of psychotropic medication and a preference for admission to a respite house over hospital admission, the combined model would yield that the treatment team have a strong reason to support Daniel in finding a respite house as soon as possible.

When the aforementioned strategies are used by appeal to PADs, it is essential that debriefing takes place afterward. This gives the treatment team the opportunity to explain why the interventions have been used, and it gives service users the opportunity to say whether they find the chosen intervention appropriate. It is recommendable to involve the support person or substitute decision maker in this conversation if service users approve of this. If service users have no support person or substitute decision maker, they can be offered the opportunity to appoint one. PADs should be updated based on the outcome of this conversation.

## Clinical Implications of the CRPD Model

Like the combined supported decision making model, the radical CRPD model yields that support must be provided in both vignettes. We have described various forms of decision support in the previous section. We have assumed, for the sake of the argument, that supported decision making only marginally enhances Daniel’s and Debby’s decision making capacities. Unlike the combined model, the radical CRPD model rejects the concept of competence and denies that there is a threshold of functional decision making capacities below which substitute decision making should be effectuated: the current preferences of service users should be respected, whatever their functional decision making capacity. If no concessions are made, the radical CRPD model yields unequivocal judgments on the vignettes: Daniel’s refusal of the medication for which he expressed a preference in his PAD should be respected, and the same holds for Debby’s choice to see her ex-husband Jason. The radical CRPD model would thus render both PADs ineffective.

However, even proponents of the radical CRPD model admit that concessions must be made in some hard cases. It is sometimes said that hard cases make for bad law, but in psychiatry hard cases are not marginal or highly exceptional cases. Responding to cases of self-harm, Flynn and Arstein-Kerslake concede that an individual’s current preferences “not necessarily represent the true will and preferences of an individual” (53). In such situations, PADs could first of all be used as a means to gently remind service users, as it were, of their deeply held values and commitments. Given the nature of mental health crises, the prospect of success for such gentle reminders seems limited. Flynn and Arstein-Kerslake note, however, that the right to legal capacity is limited by other legal rights and duties. Accordingly, they hold that support persons are permitted to act against the current preferences of a person only if the two following conditions are satisfied: “the support person is acting in an emergency situation and [ … ] supporting the person’s wishes would constitute civil or criminal negligence” (53).

Let us return to the vignettes to derive recommendations. It would seem that in neither case the two conditions are satisfied. A support person in Debby’s case would clearly not be in an emergency situation, especially given that Arstein-Kerslake and Flynn stress that necessity defenses “need to be extremely limited” (53). The support person would thus have to give effect to Debby’s current preferences on Flynn and Arstein-Kerslake’s model, and this amounts to rendering her PAD ineffective. Stretching the notion of emergency somewhat, it could be argued that a support person in Daniel’s case would be in a situation that qualifies for a necessity defense. Even then, however, it is very unlikely that respecting Daniel’s current preferences (i.e., allowing him to go home without medication) would constitute civil or criminal negligence under current laws—and arguably this is even more unlikely under CRPD-compliant laws. The support person must thus give effect to Daniel’s current preferences on Flynn and Arstein-Kerslake’s model, and this amounts to rendering his PAD ineffective.

Moreover, even if we were to assume that supporting Daniel’s current preferences would constitute civil or criminal negligence, the model would not allow mental health professionals to treat Daniel in accordance with his PAD instruction, which is to be given olanzapine or quetiapine rather than typical antipsychotic medication. The reason is that, in keeping with the logic of necessity defenses, Flynn and Arstein-Kerslake hold that involuntary interventions “should never rise to the level of forced medical or psychiatric treatment” (53).

Proponents of the radical CRPD model might admit that their model renders ineffective PAD instructions that deviate from current preferences yet claim that it does not render ineffective PAD instructions that overlap with current preferences. After all, if Daniel’s PAD had contained a general refusal of all psychotropic medication, the model’s clinical recommendations would be in line with Daniel’s PAD instructions. Two things can be said in response. First of all, the empirical evidence reviewed at the beginning of this article showed that service users typically use PADs to express preferences among treatment options and that PADs documenting general refusals of psychiatric treatment are highly exceptional. The radical CRPD model would thus render the bulk of PADs ineffective. Second, even if we were to assume that Daniel’s PAD contained a general refusal of psychiatric treatment, the model’s recommendation to withhold treatment would not be based on Daniel’s PAD but on his current preferences. Indeed, the model would yield the very same recommendation if Daniel had never completed a PAD. The PAD makes no difference.

Bach and Kerzner propose a more realistic and flexible version of the CRPD model (54). Where Flynn and Arstein-Kerslake completely reject the functional approach to competence, Bach and Kerzner propose to use a functional competence assessment to determine an individual’s legal status. Individuals are accorded what Bach and Kerzner call “legally independent” status only if their functional decision making capacities meet a certain threshold. When an individual’s capacities fall below this threshold, the individual can still exercise legal capacity through a supported decision making status when there is “at least one other person who has personal knowledge of the individual [and who] can reasonably ascribe to the individual’s actions, personal will and/or intentions consistent with the person’s identity, and can take reasonable consequential actions to give effect to the will and/or intentions of the individual” (54). Thus, if it is reasonable to assume that Daniel’s and Debby’s PADs instructions are more consistent with their identities (or own conceptions of the good) than their current preferences, Bach and Kerzner’s model allows support persons to take reasonable consequential actions to give effect to the PADs. Plausibly, these actions are precisely those that substitute decision makers are permitted to take under the combined supported decision making model.

In view of this, the combined supported decision making model and the flexible CRPD model proposed by Bach and Kerzner seem to converge: just like the combined model, the flexible CRPD model uses a functional competence assessment to determine service users’ legal status, and the responsibilities that the flexible CRPD model assigns to support persons under a supported decision making arrangement are the same as those that the combined model delegates to substitute decision makers under a substituted decision making arrangement. The only difference seems to be that proponents of each model call things by a different name. Disputes between proponents of the two models thus appear merely verbal.

An important difference must be noted, however. Since on the flexible CRPD model a service user in a supported decision making status still has legal capacity, consequential actions taken by a support person will count as an exercise of the service user’s legal capacity. The concern has been raised that supported decision making may turn into *de facto* substituted decision making (55–57) and that this will not only render service users more susceptible to undue influence but also make it more difficult to make support persons accountable for their actions (8). Efforts have been made to conceptualize supported decision making and to address concerns about undue influence and accountability (53, 58–61). We believe that the combined supported decision making model can address these concerns because it combines the virtues of non-arbitrariness and transparency. The combined model is non-arbitrary because it determines service users’ legal status by means of competence assessments, which yield very high levels of interrater reliability. The combined model is transparent because it makes explicit that service users decide for themselves and exercise their legal capacity as long as they are under a supported decision making arrangement and that substitute decision makers decide on behalf of service users under a substituted decision making arrangement.

## Conclusion and Actionable Recommendations

Mental health crises can disrupt the life plans of service users. PADs enable service users to remain in control of their life and treatment, and most service users complete PADs with this aim in mind. Various UN human rights bodies see PADs as a valuable form of support, but we have shown that the radical CRPD model adopted by some of these bodies renders PADs ineffective in situations where they could be of most use to service users. The clinical recommendations that follow from a more realistic and flexible CRPD model differ less from competence-based models than is often assumed, though concerns remain about undue influence and the accountability of support persons who give effect to PAD instructions. A model that combines supported decision making with competence assessment can address these concerns adequately.

There is enough common ground between the combined supported decision making model and the flexible CRPD model for proponents of each model to support the following recommendations:

- Policy makers should make legal provisions for PADs and limit mental health professionals’ legal leeway in overriding PADs
- Mental health professionals should actively offer service users the opportunity to complete a PAD and support them in the process of completion
- PADs should be stored in ways in which accessibility to mental health professionals in crisis situations is ensured
- Competence assessments provide non-arbitrary criteria based on which it can be decided whether support persons should support service users’ current preferences or give effect to PAD instructions
- Supported decision making must be provided before competence is assessed
- Mental health professionals must consult PADs in crisis situations and honor advance treatment refusals and requests
- All less restrictive alternatives must be exhausted before the option of involuntary treatment is contemplated
- Debriefing should be initiated after treatment has been provided based on a PAD, and PADs must be updated in light of this conversation

The implementation of these improvements in policy and practice will be an important step toward ensuring the equal treatment and promoting the autonomy of mental health service users.

## Author Contributions

All authors made substantial contributions to the conception and design of the work. AG and MS reviewed the empirical literature, MS reviewed the reports of the relevant UN bodies, and JG and MS constructed the clinical vignettes. MS worked out the central arguments and prepared the various drafts of the manuscript. AG, JG and JV revised the drafts critically for important intellectual content. All authors agree with the paper’s arguments and conclusions and gave approval for the final version to be published.

## Funding

This research is part of the project SALUS (2018–2024) and is supported by a grant from the German Federal Ministry of Education and Research (grant number 01GP1792).

## Conflict of Interest Statement

The authors declare that the research was conducted in the absence of any commercial or financial relationships that could be construed as a potential conflict of interest.
